# A Model for Predicting and Grading the Quality of Grain Storage Processes Affected by Microorganisms under Different Environments

**DOI:** 10.3390/ijerph20054120

**Published:** 2023-02-25

**Authors:** Qingchuan Zhang, Zihan Li, Wei Dong, Siwei Wei, Yingjie Liu, Min Zuo

**Affiliations:** National Engineering Research Centre for Agri-Product Quality Traceability, Beijing Technology and Business University, Beijing 100048, China

**Keywords:** grain quality change prediction, grain, prediction, FEDformer

## Abstract

Changes in storage environments have a significant impact on grain quality. Accurate prediction of any quality changes during grain storage in different environments is very important for human health. In this paper, we selected wheat and corn, which are among the three major staple grains, as the target grains whose storage monitoring data cover more than 20 regions, and constructed a grain storage process quality change prediction model, which includes a FEDformer-based grain storage process quality change prediction model and a K-means++-based grain storage process quality change grading evaluation model. We select six factors affecting grain quality as input to achieve effective prediction of grain quality. Then, evaluation indexes were defined in this study, and a grading evaluation model of grain storage process quality was constructed using clustering model with the index prediction results and current values. The experimental results showed that the grain storage process quality change prediction model had the highest prediction accuracy and the lowest prediction error compared with other models.

## 1. Introduction

Fusarium and Aspergillus are the main pathogenic fungi of grain and oil crops [1,2]. In warm and humid areas, serious diseases will reduce the quality of crops such as wheat and corn, posing a serious threat to grain and oil production. Once the temperature and humidity of grain storage environments change, it is susceptible to pathogenic fungi and molds, and some may also produce mycotoxins, such as aflatoxin B1 (AFB1) produced by Aspergillus flavus and Fusarium. This fungus produces deoxynivalenol (DON) and zearalenone (ZEN). Therefore, a suitable environment is very important to ensure the quality of grains during storage, and experiments by Zain et al. [3] demonstrated that temperature and moisture are important factors affecting toxin derivation. Among others, it is important to calculate the equilibrium moisture content in order to understand the behavior of moisture content of grains in storage environments [4]. In addition, aflatoxin B1 (AFB1), deoxynivalenol (DON) and zearalenone (ZEN) are common and important toxins in wheat and maize, which pose serious risks to grain quality and human health. Other common non-toxic fungi rarely exceeded the limit values in historical testing records; therefore, in this paper, toxic fungi were selected as the main factors when conducting the quality prediction and constructing grading model of grain storage process affected by microorganisms in different environments. On this basis, temperature and moisture were selected as environmental variables, and aflatoxin B1 (AFB1), deoxynivalenol (DON) and zearalenone (ZEN) were chosen as monitoring indicators for this experiment and sampled and tested regularly.

As the world economy develops, and as people’s living standards continue to improve, food production increases year by year and food quality issues are of increasing concern. It is noteworthy that global post-production grain quantity and quality losses due to harvesting, storage, deterioration, and insect and mold contamination account for 15% to 20% of the total. Grain storage suffers from serious quality losses, the main causes of which include moderate reduction, dry matter depletion, and pest infestation. Therefore, reducing food losses due to storage and improving food utilization and safety are urgent needs that must be addressed internationally. This is an important prerequisite for establishing a resilient and developing a sustainable global agricultural food system.

In the face of such complex challenges, scholars have conducted research on predicting and evaluating quality changes during grain storage in order to determine the appropriate environmental settings, both to improve grain storage quality and to reduce grain quality losses during the storage phase. Coradi et al. [1] developed six linear regression models to predict grain storage quality and evaluated the models to achieve high prediction accuracy. Faree et al. [2] used multiple linear regression and the artificial neural network (ANN) to predict the quality of maize grains during storage; they achieved better prediction results. Lutz et al. [5] used a wireless sensor network, an IoT platform, to monitor the equilibrium moisture content in real time and used ANN to predict the quality of maize grains stored under different conditions. Szwedziak et al. [6] used a proprietary computer application based on the RGB model to assess the contamination status of maize grains. Xie et al. [7] predicted public risk perceptions more accurately by building bp neural networks. Liu et al. [8] constructed a bidirectional long- and short-term memory (BiLSTM) model and selected six influencing factors of municipal solid waste power generation as input indicators to achieve an effective prediction of municipal solid waste power generation.

In the current research, deep learning methods have been gradually applied to the prediction of quality changes in grain storage processes, but because of the close dependence of any quality changes in grain storage processes on environmental factors such as temperature and humidity with temporal characteristics, simple artificial neural networks (ANNs) cannot solve the problem of gradient explosion and information distortion, and their prediction accuracy is often lower than that of deep learning that can learn the close dependence of methods.

In this study, we developed a FEDformer-based prediction model for quality changes in grain storage process and a K-means++-based grading evaluation model for quality changes in grain storage process. Firstly, in the prediction model, we use three factors affecting grain quality to predict the grain quality changes during storage to reduce the uncertainty of the prediction model. Secondly, in the clustering model, we set an evaluation index S based on the conclusion of the prediction model, which integrates the current and predicted values of toxin content to grade and evaluate the quality changes during grain storage. The experimental results showed that the grain storage process quality change prediction model had the highest prediction accuracy and the lowest prediction error compared with other models. Finally, we suggest corresponding suggestions for optimizing grain storage.

The contributions of this study include three main aspects: (1) the establishment of a FEDformer-based model for predicting quality changes in grain storage process. Experiments show that the model is more accurate in predicting the quality changes of grain, as compared with several other deep learning models. (2) The establishment of a grading evaluation model based on K-means++ for the quality change of grain storage process. Based on the experimental results of this model, a reasonable grading evaluation of grain quality can be obtained. (3) The analysis, based on the changes of toxin content in grain during storage and grain quality grading evaluation obtained from the above study, of the factors influencing the quality changes in grain storage process by microbial environment. Corresponding suggestions are made for the optimization of grain storage. In addition, the environmental and quality changing data in the process of grain storage provide support for the subsequent blockchain-based grain collection, storage and transportation whole process traceability [9,10].

The structure of the paper is as follows: Section 2 reviews the previous literature. Section 3 presents the prediction model and the clustering model proposed in this paper. Section 4 describes the experimental results and analysis. Section 5 is the discussions and implementations section. Finally, the paper is concluded.

## 2. Literature Review

### 2.1. Factors Affecting the Quality of Wheat and Corn during Storage

According to the survey, there are many factors that affect the quality of wheat and corn during storage, the most important of which are toxins [1,2]. Grain contamination by mycotoxins such as deoxynivalenol (DON), aflatoxin B1 (AFB1), and zearalenone (ZEN), which are very stable and are not metabolized, is harmful to humans and animals [11,12]; grain contamination by mycotoxins has been observed even in the absence of yield reduction, thus leading to yield loss [13]; furthermore, several of these toxins are highly toxic to both humans and animals, depending on the type of toxin and the amount of food or feed consumed. Consumption of food or feed contaminated with mycotoxins can cause various diseases in humans and animals, generally known as mycotoxicosis, with strong toxic effects such as skin irritation, vomiting, diarrhea, weakness, loss of appetite, bleeding, neurological disorders, abortion, and may even produce death [14]. In addition, a study by Bennett et al. [11] showed that some types of Fusarium toxins (zearalenone ZEN, etc.) are associated with an increasing number of cancers in humans. Therefore, toxins in grain seriously affect grain quality and endanger human health [15], which must be paid sufficient attention. Toxin production in grain is a complex process, and the rapid reproduction and growth of microorganisms are responsible for its toxicity [6,16], while the growth of microorganisms is closely related to the environment and is mainly influenced by temperature and moisture [17,18]. Toxin-producing fungi in microorganisms originate mainly from various fungi of the genera Aspergillus, Penicillium and Camara; these fungi are capable of producing various toxic secondary metabolites such as aflatoxin B1, zearalenone ZON and deoxynivalenol DON, which lead to an accelerated respiration rate of grain quality, increasing the explanation of carbohydrates, proteins and oils, thus seriously affecting the quality of grain [4].

Storage conditions such as a climate suitable for toxin growth, moisture, temperature and oxygen levels are considered as influencing factors for toxin production [19,20]. Among them, temperature and moisture are the key factors affecting microbial growth [3], both of which affect grain quality by influencing the activity of grain microorganisms. In a study by Saleemullah et al. [21], aflatoxin content was measured and analyzed in grains stored for 18 months, and the aflatoxin content increased from 27.1 L/kg to 31.9 L/kg; this indicates that the aflatoxin content of grains was strongly influenced by the storage period, and subsequent experiments showed that storing grains in warehouses during heavy rains led to increased formation of toxins such as aflatoxins. Kumar et al. demonstrated that high temperatures are considered to be an important determinant of fungal growth and production of toxins such as AFB1 [22]. 

The activity of microorganisms is closely related to the environment in which they live; any change in the environment will affect their activity. Suitable environmental conditions can promote the growth and reproduction of microorganisms, while adverse environmental conditions can inhibit the growth of microorganisms and can even cause their death. The impact of moisture on wheat and corn in storage is manifested in this way: once in a water content environment suitable for microbial growth, wheat and corn are susceptible to mold infiltration by plant pathogens and produce fungal toxins, such as aflatoxin, zearalenone, vomitoxin, etc., thus affecting their quality. The influence of temperature on the storage process of wheat and maize is mainly manifested in the fact that the change in grain temperature is closely related to the condition of the grain itself, microbial activity, and many other factors. 

Baliukoniene et al. [19] conducted experiments to determine toxins in maize and wheat at different storage temperatures; when the temperature in the silo was 15–25 °C and after one month of storage, wheat was strongly contaminated with micro fungi: wheat contained 31.37 × 103 cfu/g, which was 50% and 71% higher compared to other grain bins at different temperatures, and zearalenone ZEN content was 2.89 µg/kg in corn and 5.01 µg/kg in wheat. In contrast, the zearalenone ZEN content of corn and wheat in other grain silos ranged from 40% to 64.6% of the levels mentioned above. The effect of grain temperature on grain storage quality is mainly expressed through the effect on pests, microorganisms. and grain quality. Food security storage and the maintenance of grain quality can be achieved by controlling the temperature of the environment in which the grain pile organisms are located, limiting the growth and reproduction of harmful organisms, and delaying the aging of grain quality.

### 2.2. Overview of Prediction Methods

The time series forecasting methods in existing studies can be classified into traditional linear regression methods [23,24], machine learning methods [25,26], and deep learning methods [27,28,29,30,31,32,33,34,35,36,37,38,39,40]. Traditional linear regression methods are moving average models (MA) based on historical white noise modeling, autoregressive models (AR) based on historical time series modeling, and autoregressive moving average models (ARIMA) that combine the first two models. These are also widely used in time series forecasting tasks [23,24]. However, none of the above models can capture nonlinear relationships. Machine learning can solve simple non-linear relationships. Drucker et al. [25,26] equipped a support vector machine (SVM) with regression prediction capability for nonlinear data by introducing soft interval and distance loss functions, hence the name support vector regression (SVR). Yu et al. used a two-stage support vector regression (BI-SVR) based on Bayesian inference to predict a feeding batch of penicillin culture process with the performance of soft sensors, and the prediction results of BI-SVR were significantly improved compared to SVM. Jaques et al. [41] used the decision tree algorithms REPTree and M5P, random forest and linear regression for predicting the physical and physiological quality of soybean seeds and experimentally demonstrated an improved accuracy index compared to linear regression. 

Compared with the above methods, deep learning methods are able to solve more complex nonlinear problems. Artificial neural networks (ANN) mainly consist of the input, hidden, and output layers, with multilayer perceptron (MLP) being the most commonly used. Asadollahfardi et al. [27] applied ANN in the MLP framework to predict total dissolved solids (TDS) in Zayande Rud River, Isfahan Province, Iran, and could obtain more reliable prediction results. Recurrent neural networks (RNN) [28,29] are capable of learning dynamic temporal features using memory units, but the model suffers from the problem of being prone to gradient disappearance and difficulty in learning long time dependencies. The long short-term memory network (LSTM) [30,31,32] solves the problem of the gradient disappearance of longer sequences in training by learning temporal dependencies through a gate mechanism; it can maintain temporal information in the state for a long time, and it is widely used in time series prediction. Kang et al. [33] and Vo et al. [34] applied the bidirectional long short-term memory network Bi-LSTM to time series prediction. Compared to LSTM, it considers both forward and backward sequences, which is advantageous for time or location data with contextual compliance features. The selected pass recurrent unit (GRU) [35,36] is a simplified version of LSTM; it combines forgetting and input gates as update gates, and it has fewer parameters and reduced complexity compared to LSTM. Yang et al. [37] proposed a BRNN-based method for predicting the remaining time of tram charging, Bi-GRU, in order to solve the problem of one-way prediction; compared to LSTM and SVR models, it performed better in terms of accuracy and stability. In 2017, Vaswani et al. [38] proposed a novel architecture Transformer, which showed powerful modeling capabilities for long-term dependencies and interactions in time series data.

Zhang et al. [39] used the Transformer-based time series prediction model for predicting the next hour’s electricity consumption and achieved promising results. Many Transformer variants have been proposed to address the special challenges in time series forecasting tasks. Among them, Zhou et al. [40] proposed the Informer framework, which is an efficiency optimized long time series forecasting model based on the Transformer; it greatly reduces the time complexity and the space complexity of the Transformer.

## 3. Methodology and Study Area

In recent years, Transformer [42] has become a typical representative of neural network models used in the field of time-series prediction. FEDformer [43] is an improved model based on Transformer; it focuses on the implementation of analyzing the relationship characteristics between data indicators, reducing the time complexity, improving the prediction accuracy and model learning efficiency of indicators, and thus reasonably and effectively predicting toxin content.

### 3.1. Data Source

Grain storage monitoring data for this study covered more than 20 regions with 139 wheat and corn samples totaling 2100 units of data, with wheat and corn originating from the middle and lower temperate regions. The datasets for training and testing in the experiment were divided as shown in Table 1.

In addition, wheat and maize were obtained from the middle and lower reaches of temperate river valley production areas. The microbial toxin limits selected for this paper were as follows: 20 μg/kg for aflatoxin B1, 500 μg/kg for zearalenone ZON and 1000 μg/kg for deoxynivalenol DON for maize; 5 μg/kg for aflatoxin B1, 60 μg/kg for zearalenone ZON and 1000 μg/kg for deoxynivalenol DON for wheat. Fusarium DON was limited to 1000 μg/kg.

### 3.2. FEDformer-Based Model for Predicting Quality Changes in Grain Storage Processes

#### 3.2.1. Model Fundamentals

FEDformer combines Transformer and seasonal-trend decomposition [44] methods, capturing the global pattern of the world sequence with the seasonal-trend decomposition method, while capturing the more detailed structure with Transformer FEDformer’s. The main structure (backbone) uses an encoder–decoder structure, consisting of n encoders and m decoders, and it includes four internal submodules: a frequency domain learning module (Frequency Enhanced Block), a frequency domain attention module (Frequency Enhanced Attention), a period trend decomposition module (MOE Decomp), and a one-dimensional Convolution module (Conv1d).

The MOE Decomp module decomposes the sequence into a periodic term (seasonal, S) and a trend line (trend, T). This decomposition is not performed only once, but in an iterative decomposition mode. In the encoder, the input passes through two MOE Decomp layers, each of which decomposes the signal into two components: seasonal and trend. The trend component is discarded, and the seasonal component is passed to the next layers for learning and finally to the decoder. In the decoder, the input of the encoder also passes through three MOE Decomp layers and is decomposed into seasonal and trend components. Among them, the seasonal component is passed to the next layers for learning, where the frequency domain Attention (Frequency Enhanced Attention) layer learns the frequency domain correlation between the seasonal term of the encoder and the decoder, and the trend component is summed up and finally added back to the seasonal term to restore the original sequence.

Among the Frequency Enhanced Block (FEB) and the Frequency Enhanced Attention (FEA), the Attention mechanism used in the Frequency Enhanced Attention (FEA) is of linear complexity, while the Attention mechanism used in the traditional Transformer is of square complexity. This has the advantage of greatly reducing the length of the input vector and thus the computational complexity, but this sampling must be detrimental to the input information. This loss must be detrimental to the input information. However, experiments have shown that this loss has little impact on the final accuracy. This is because the general signal is sparser in the frequency domain compared to the time domain. Moreover, a large amount of information in the high frequency part is so-called noise; it can often be discarded in time series prediction problems, since noise often represents a randomly generated part and thus cannot be predicted. 

**Figure 1:**
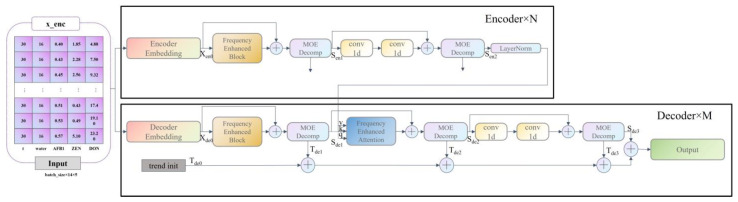
FEDformer Model.

In the learning phase, FEB uses a fully concatenated layer R as a learnable parameter. FEA, on the other hand, performs a cross-attention operation on the signals from the encoder and decoder in order to learn the intrinsic relationship between the two parts of the signal. The frequency domain complementation process is relative to the previous frequency domain sampling. In order to cause the signal to revert to its original length, the frequency points not picked by the previous sampling need to be zeroed and projected back to the time domain, because the signal projected back to the frequency domain is the same as the previous input signal dimension by the complementation operation in the previous step. The FEDformer model is shown in Figure 1.

The specific function of the encoder is shown in the following Equations:

(1) $$S_{en1},\_=MOEDecomp\left(FEB\left(X_{en0}\right)+X_{en0}\right),$$

(2) $$S_{en2},\_=MOEDecomp\left(conv1d\left(conv1d\left(FEB\left(X_{en0}\right)+X_{en0}\right)\right)\right).$$

The specific function of the decoder is expressed in Equations

(3) $$S_{de1},T_{de1}=MOEDecomp\left(FEB\left(X_{de0}\right)+X_{de0}\right),$$

(4) $$S_{de2},T_{de2}=MOEDecomp\left(FEA\left(S_{de1},LayerNorm(S_{en2}\right)+S_{de1}\right)),$$

(5) $$S_{de3},T_{de3}=MOEDecomp\left(conv1d\left(conv1d\left(S_{de2}\right)\right)+S_{de2}\right).$$

#### 3.2.2. Model for Predicting Quality Changes during Grain Storage

Our data are divided into six dimensions of information with a period of 30 days; these are time, temperature, moisture content, AFB1 content, ZEN content, and DON content, where AFB1, ZEN, and DON content are predictors. Therefore, to be applicable to the application scenario of this paper, we improved the construction of the model Encoder embedding as well as that of the Decoder Embedding.

**Figure 2:**
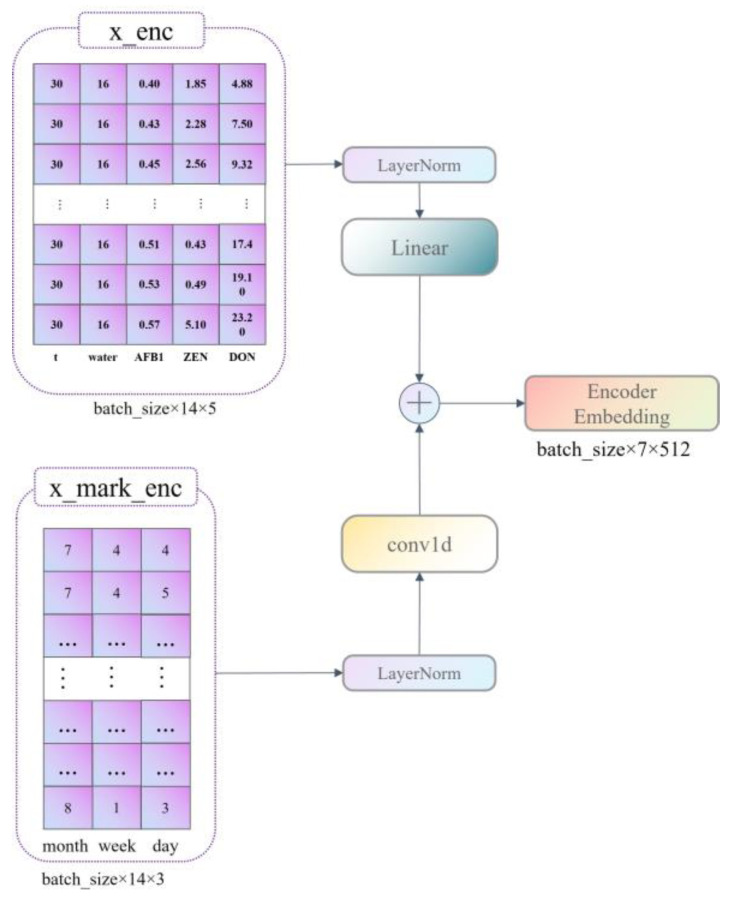
Encoder Embedding Construction Flowchart.

**Figure 3:**
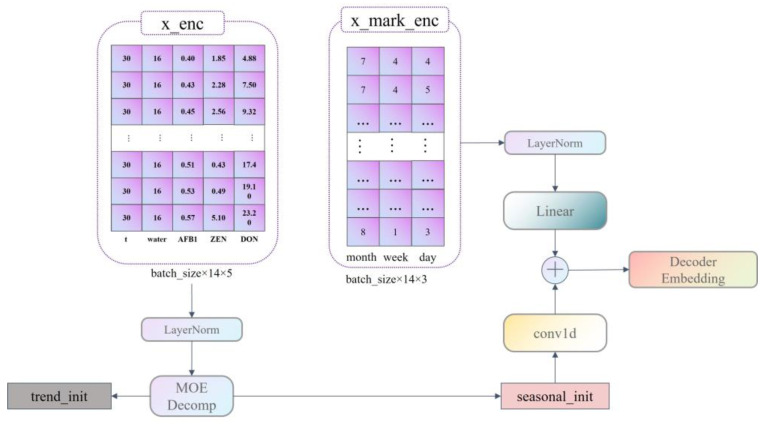
Decoder Embedding Construction Flowchart.

First, we set the three dimensions of month, week, and day to represent the characteristics of the time dimension; this has the advantage of replacing the time dimension from one-dimensional information to three-dimensional information, and it can correctly represent the time sequence information and enhance the importance of the time dimension, making the model pay more attention to the characteristics of the time dimension in the learning process so as to predict the indicators more effectively. The construction of Encoder embedding is shown in Figure 2. The construction of the decoder embedding is shown in Figure 3.

**Figure 4:**
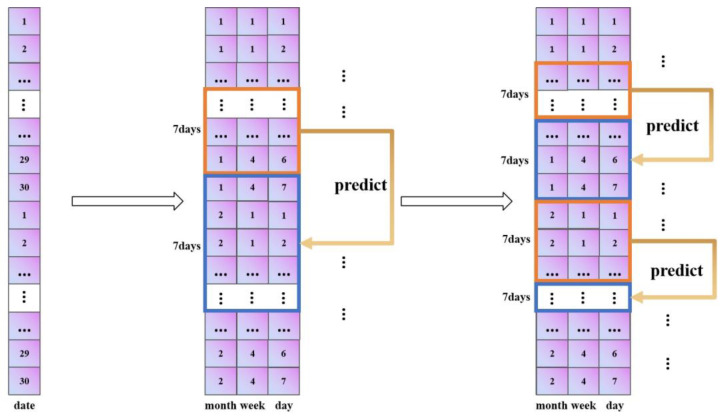
Improvement flow chart.

Second, in the model, we change the data reading method from sequential reading to reading with a 30-day period; this prevents the situation in which different samples predict each other, and thus it reasonably applies to the scenario in this paper. Finally, we set the data of the first seven days to predict the data of the next seven days. The specific improvement process is shown in Figure 4.

### 3.3. Grading Evaluation of Quality Changes in Grain Storage Process Based on K-Means++

In order to evaluate the grade of quality changes in the grain storage process in grain silos, we set an evaluation index *S*, which integrates the current and predicted values of toxin content, and the formula of the evaluation index *S* is shown in Equation (6).

(6) $$S=\left[y_{i},{\bar{y}}_{i}\right],$$

where $y_{i}$, i∈{1,2,…, *n*} is the true value, ${\bar{y}}_{i}$, i∈{1,2,…, *n*} is the mean of the predicted values in the next 7 days, and n is the number of indicator variables.

In this paper, a clustering algorithm is used to grade all samples for quality variation and to construct a quality grading space based on the evaluation index *S*. Since the amount of data in this subject is small and there are no dirty data, the K-means++ algorithm is fast and efficient, and it can achieve good clustering performance on the sample space of arbitrary shape, which is suitable for analyzing the model data of this study, so the K-means++ algorithm is selected for the grain quality change grading in this paper. The K-means++ algorithm is an improvement of the K-means algorithm, and its main difference is the initialization to determine the initial clustering center.

**Figure 5:**
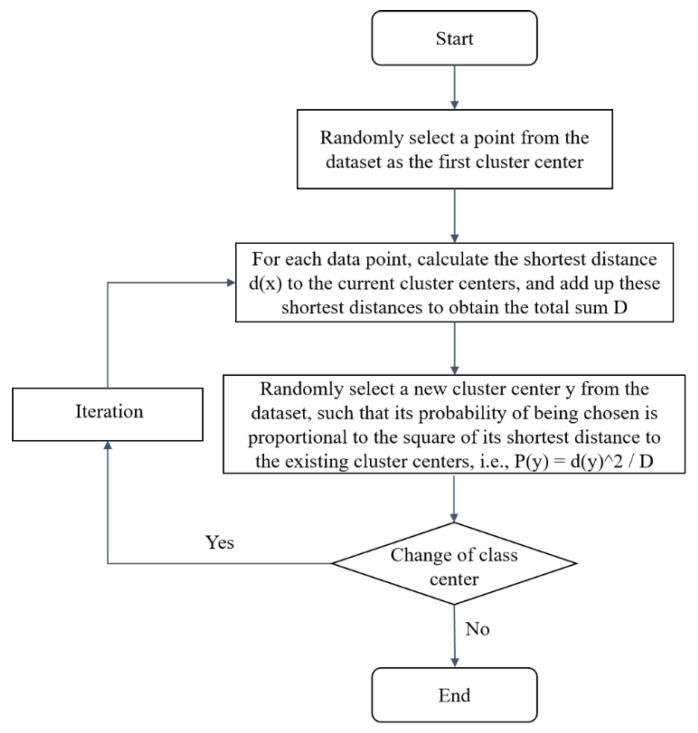
K-means++ algorithm flow chart.

K-means algorithm determines the initial clustering center randomly, while K-means++ is based on the distance from the current sample point to the existing center point to provide the probability of the sample point’s becoming the next clustering center (the greater the distance, the greater the probability), and then, according to the probability size, to extract the next clustering center, and to repeat until the extraction of K clustering centers. The specific steps are shown in Figure 5.

### 3.4. Model Evaluation Metrics

#### 3.4.1. Evaluation Metrics for Predictive Models

The evaluation metrics of the prediction models are the mean absolute percentage error (*MAPE*), the mean square error (*MSE*), the root mean square error (*RMSE*), the mean absolute error (*MAE*), and the symmetric mean absolute percentage error (*SMAPE*), respectively; they are used to evaluate the prediction performance and the degree of fit of the models. *MAE*, *MSE*, *RMSE*, *MAPE*, and *SMAPE* are used to measure the difference between the predicted data and true data and the range of values. A perfect model is equal to zero when the predicted value exactly matches the true value; the larger the error, the larger the value.

The formula for calculating the mean absolute percentage error is shown in (7):

(7) $$MAPE=\frac{100\%}{n}\overset{n}{\underset{i=1}{\sum }}|\frac{{y^{\prime }}_{i}-y_{i}}{y_{i}}|$$

The formula for calculating the mean square error is shown in (8):

(8) $$MSE=\frac{1}{n}\overset{n}{\underset{i=1}{\sum }}{\left({y^{\prime }}_{i}-y_{i}\right)}^{2}$$

The formula for calculating the root mean square error is shown in (9):

(9) $$RMSE=\sqrt{\frac{1}{n}\overset{n}{\underset{i=1}{\sum }}{\left({y^{\prime }}_{i}-y_{i}\right)}^{2}}$$

The formula for calculating the mean absolute error is shown in (10):

(10) $$MAE=\frac{1}{n}\overset{n}{\underset{i=1}{\sum }}|{y^{\prime }}_{i}-y_{i}|$$

The formula for calculating the symmetric mean absolute percentage error is shown in (11):

(11) $$SMAPE=\frac{100\%}{n}\overset{n}{\underset{i=1}{\sum }}\frac{|{y^{\prime }}_{i}-y_{i}|}{\left(|{y^{\prime }}_{i}|-|y_{i}|\right)/2}$$

where $y_{i}$, i∈{1,2,…, *n*} is the true value, $y_{i}^{\prime }$, i∈{1,2,…, *n*} is the predicted value, and *n* is the number of indicator variables.

#### 3.4.2. Evaluation Metrics for Clustering Models

The evaluation index of the clustering model is the contour coefficient *S*. The core idea of the contour coefficient is to determine the relative size of the inter-class distance and intra-class distance. The value is between [−1,1], and the larger the value, the better the clustering result. The formula for the profile coefficient *S* is shown in (12):

(12) $$S=\frac{1}{N}\overset{N}{\underset{i=1}{\sum }}\frac{b\left(i\right)-a\left(i\right)}{max\left\{a\left(i\right),b\left(i\right)\right\}}$$

where $a\left(i\right)$ is the average distance of other samples in the cluster to which i belongs, $b\left(i\right)$ is the minimum value of the average distance of samples from *i* to other clusters, and *N* is the number of samples.

## 4. Results

### 4.1. Comparative Experiments of Models for Predicting Quality Changes during Grain Storage

In order to effectively evaluate the performance of the FEDformer-based model in predicting the quality changes during grain storage, in this paper, several deep learning prediction methods are selected as comparison experiments, and we set up a 5-fold cross-validation experiment in this experiment in order to prevent overfitting.

In addition, the FEDformer model contains several hyperparameters that affect the accuracy of the model, and we determined that the learning rate and the number of days to predict the future have the greatest impact on the performance of the model through experiments, and conducted several comparative experiments for this purpose. Each parameter setting in the proposed model is shown in Table 2.

Among the eight prediction models, CNN has the largest prediction error, while the traditional LSTM is second only to CNN. In addition, four prediction models, LSTM, GRU, BILSTM and BIGRU, have similar prediction accuracy and little difference in prediction error. Transformer, Informer, and FEDformer have significantly higher prediction accuracy and lower prediction error than other prediction models. The prediction error is significantly reduced. Compared with other models, the prediction error of FEDformer is the smallest, and the experiments of MAE, MSE, RMSE, MAPE, and MSPE of FEDformer model for the maize test set are 0.01, 0.0005, 0.023, 0.01, and 0.09, respectively. The results are shown in Table 3.

The MAE, MSE, RMSE, MAPE and MSPE of FEDformer for the wheat test set were 0.017, 0.0006, 0.025, 0.108, and 0.14, respectively. The experimental results for MAE, MSE, RMSE, MAPE, and MSPE for the wheat test set are shown in Table 4.

### 4.2. Comparison of Clustering Models for Quality Changes during Grain Storage

**Figure 6:**
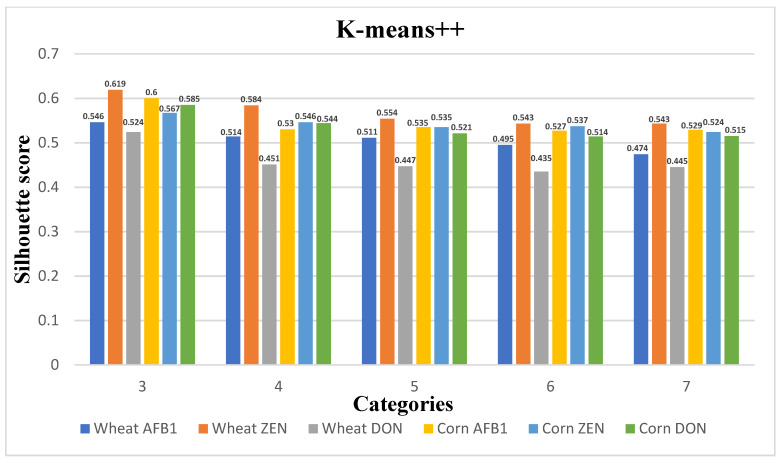
K-means++; 5 types of clustering category profile coefficients.

**Figure 7:**
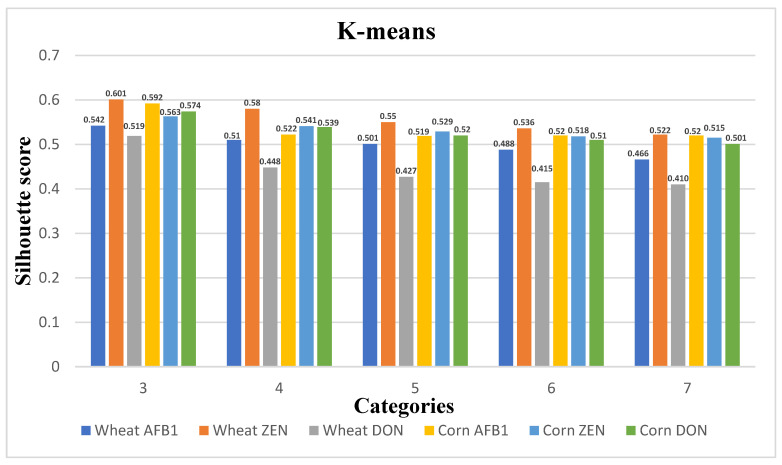
K-means; 5 types of clustering category profile coefficients.

**Figure 8:**
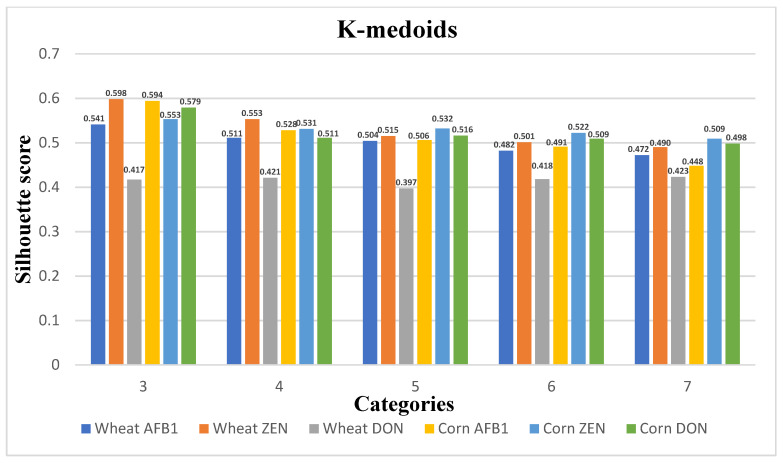
K-medoids; 5 types of clustering category profile coefficients.

In this paper, the clustering algorithm selects the S-value of the evaluation index for each sample per day as the clustering feature. Figure 6, Figure 7 and Figure 8 show the plots of the clustering results of K-means++, K-means, and K-medoids for wheat and maize with their corresponding three toxins, respectively, showing the line graphs of the number of clusters from 3–7 contour coefficients for wheat and maize. From the figure, the contour coefficients of 3–7 clusters of the three clustering models basically show a decreasing trend, and the contour coefficients of three clusters are the largest, indicating compactness between instances within three clusters and large inter-cluster distances. 

In addition, the contour coefficient of the K-means++ clustering model is the maximum among the three models, so the K-means++ clustering model is selected as the clustering model for quality change in grain storage process in this paper. The grain quality change is divided into three levels, and the cluster centers, grain quality change, and the number of samples in each level for the three toxin levels corresponding to wheat and corn are shown in Table 5 and Table 6. The distance of the cluster centers from the origin was calculated based on the indicators, and the categories 1–3 were defined as grain quality variation levels1-level2-level3, respectively. from which the indicators of the cluster centers increase sequentially with the increase of the quality levels.

Figure 6 plots the clustering results of K-means++ for wheat and maize with their corresponding three toxins, and the results show that the three clusters have the largest profile coefficients and large distances between clusters, thus classifying the grain quality variation into three classes.

Figure 7 plots the clustering results of K-means for wheat and maize with their corresponding three toxins. Compared with K-means++, the contour coefficients of each cluster are generally lower than the results of K-means++, and the contour coefficients of three clusters are still the maximum.

Figure 8 plots the clustering results of K-medoids for wheat and maize with their corresponding three toxins. Compared with K-means++ and K-means, the contour coefficients of each cluster are generally lower than the results of the above two algorithms, and the contour coefficients of three clusters are still the maximum.

Table 5 shows the clustering centers, the grain quality variation, and the number of samples in each level for the three toxin levels corresponding to wheat. The results showed that most wheat samples had zearalenone ZON and deoxynivalenol DON levels in Level 1, while most wheat samples had aflatoxin B1 levels in Level 2.

Table 6 shows the clustering centers, the grain quality variation, and the number of samples in each level for the three toxin levels corresponding to maize. The results showed that the vast majority of maize samples contained aflatoxin B1, zearalenone ZON, and deoxynivalenol DON at Level 1.

## 5. Discussion

Food quality is related to human health, and a decline in food quality increases the risk of human illness [13], in addition to the current global spread of COVID-19, which is impacting international food supply chains and straining food supplies [41]. Sudden outbreaks of desert locusts in some countries, superimposed on global epidemics, make conventional disaster prevention and control difficult to implement, exacerbating concerns about food quality loss and food security. Therefore, it is of great interest to predict the quality of grain during storage; however, the factors that lead to changes in quality during storage are complex, and the deterioration in grain quality is due to contamination by toxins produced by microorganisms during storage, which seriously affects people’s health, where temperature and moisture are important factors that affect microbial activity; thus, the toxin content is a decisive factor in the quality of grain, and therefore our experiment used it as a monitoring indicator and temperature and moisture content as environmental variables.

According to the experiment, we determined that the content of aflatoxin B1 (AFB1), deoxynivalenol (DON) and zearalenone (ZEN) increased with the increase in moisture content in the environment, and the content of these three toxins was highest at a moisture content of 22%, which means that 22% moisture content was most suitable for the growth of the three toxins; the three toxins also showed an increasing trend with the increase in temperature. The most suitable temperature for the growth of aflatoxin B1 (AFB1) and zearalenone (ZEN) is 25°, and the most suitable temperature for the growth of deoxynivalenol (DON) is 30°. The results of this experiment are in agreement with those of Lutz et al. [4]. In addition, we have offered some comments on the maintenance of grain quality during storage as follows.

Large losses in grain quality endanger human health, increase environmental stress, and affect the sustainable development of agricultural food. Currently, improved storage equipment is the most commonly used method to reduce food quality losses in the storage chain. A considerable number of institutions and companies around the world are using and promoting sealed storage technology, including the WFP, FAO, GrainPro, etc., by providing sealed bags and other storage equipment, thus reducing losses in the food storage chain. It is known from the results of studies that sealed bags can effectively reduce quality losses [45,46] and that they are less costly and suitable for economically underdeveloped areas. However, many times, the quality changes in grain are not detectable by the senses; this can increase the safety risks of consuming grain and can cause unpredictable adverse effects on human health. 

Our approach is based on monitoring of the storage environment and use of artificial intelligence technology to assist in decision making. The use of artificial intelligence technology not only reduces the cost of manual sampling, but also increases the sampling period. Based on our experiments, we believe that attention should be paid to the influence of environmental factors on grain quality during storage, especially temperature and moisture content, where equilibrium moisture is important [41]. The stored grain interacts with the storage conditions, the air between the grains and the storage structure, leading to variations in grain quality temperature, moisture content and relative humidity between the grains. The combination of these factors characterizes the storage environment in which the equilibrium moisture content of the grain varies.

The global market for quality grains is growing, and concerns and worries about grain quality are increasing. There is an urgent need to determine ways to reduce losses in the storage phase that will ensure food safety and agricultural sustainability. Many grain managers are investing more in efficient and reliable grain quality management technologies. Grain bin monitoring and artificial intelligence technology-assisted decision-making approaches are storage phase information systems that use information technology to ensure grain quality by controlling and monitoring environment-related factors. This is considered to be an intelligent approach that incorporates emerging information technology, and it is an efficient strategy to significantly reduce grain quality losses and labor costs, an issue that has attracted much attention worldwide. Grain bin monitoring and artificial intelligence technologies can be an option to assist decision making, and some countries have already started to invest in the development of related technologies and have put them to use. Thus, bin monitoring and AI technology-assisted decision making can play an important role in ensuring the safety and the high quality of grain.

The development of a combination of IoT-based grain bin monitoring and AI-assisted decision-making technology will be the main trend in the development of storage technology in the coming years, and monitoring technology and AI technology may further develop into mainstream applications. Since there are many factors that affect grain quality, a more accurate quantitative and qualitative evaluation is the main challenge at present. Therefore, a more accurate assessment of grain quality has important positive implications for grain utilization and safety, for economic development, and for human health.

In order to promote grain bin monitoring and AI technology-assisted decision-making methods, the implementation cost of this technology must be further explored. The ideal monitoring and AI-assisted decision-making technology should be an efficient and effective system that continuously improves grain safety and nutritional value, reduces quality losses, and addresses the international problem of grain storage losses in a sustainable manner. Therefore, a prudent policy should be adopted to enhance food safety and quality and to increase food utilization while promoting the development of information technology.

## 6. Conclusions

Environmental changes during storage are an important factor affecting the quality of grain. We determined that the levels of aflatoxin B1 (AFB1), deoxynivalenol (DON) and zearalenone (ZEN) increased with the increase in moisture content in the environment and with the increase in ambient temperature, where the environmental conditions with 22% moisture content and temperature between 25° and 30° were most suitable for the growth of these three toxins. Therefore, unfavorable environmental conditions can lead to a decrease in the quality of stored grain and an increase in toxins, which can cause significant health problems. Therefore, accurate prediction of the content of various toxins in grain under different environmental conditions and reasonable definition of quality levels can help grain managers to provide early warning of grain quality and greatly reduce the labor cost of grain toxin detection. In this study, six indicator variables were first collected, and then the contents of three toxins were input into a FEDformer-based model to predict aflatoxin B1 content based on K-means++ of grain storage process, using zearalenone content and deoxy peptide humic acid content as input variables. The evaluation index S for the current and predicted values of toxin was set, and the quality change model was constructed according to the evaluation index to evaluate the quality of grain storage process in a graded manner.

## Figures and Tables

**Table 1:** Partitioning of the dataset in the experiment.

Dataset	Training Set	Test Set	Validation Set
2100	1470	420	210

**Table 2:** Parameter setting for FEDformer-based model for predicting quality changes in grain storage processes.

Model	Learn Rate	Pred Length	RMSE	MAPE
FEDformer	0.001	3	0.028	0.14
	0.001	7	0.026	0.12
	0.001	14	0.032	0.14
	0.001	21	0.042	0.17
	0.0001	3	0.026	0.13
	0.0001	7	0.023	0.10
	0.0001	14	0.027	0.12
	0.0001	21	0.035	0.15

**Table 3:** Comparative experimental results on the performance of a model for predicting quality changes during maize storage.

Model	MAE	MSE	RMSE	MAPE	MSPE
CNN	0.20	0.05	0.24	1.00	15.00
LSTM	0.19	0.04	0.20	0.80	7.30
GRU	0.17	0.04	0.20	0.79	7.40
BILSTM	0.17	0.04	0.20	0.78	7.20
BIGRU	0.18	0.04	0.20	0.77	6.80
Transformer	0.04	0.0230	0.470	0.35	1.77
Informer	0.02	0.0006	0.025	0.12	0.20
FEDformer	0.01	0.0005	0.023	0.10	0.09

**Table 4:** Comparative experimental results of model performance for predicting quality changes during wheat storage.

Model	MAE	MSE	RMSE	MAPE	MSPE
CNN	0.19	0.05	0.23	1.05	4.10
LSTM	0.18	0.05	0.23	0.90	4.00
GRU	0.19	0.05	0.23	0.78	3.85
BILSTM	0.18	0.05	0.22	0.77	3.80
BIGRU	0.18	0.05	0.23	0.77	3.80
Transformer	0.022	0.0008	0.030	0.152	0.239
Informer	0.020	0.0007	0.029	0.138	0.219
FEDformer	0.017	0.0006	0.025	0.108	0.140

**Table 5:** Clustering centers and ranking of three clusters of wheat.

Categories	$y_{i}$	${\bar{y}}_{i}$	Sample Size	Quality Level
AFB1 1	0.18296259	0.37745829	437	Level 1
AFB1 2	0.38632914	0.51953385	479	Level 2
AFB1 3	0.59146268	0.70412027	257	Level 3
ZEN 1	0.56973928	6.38460694	574	Level 1
ZEN 2	3.72524785	9.18565112	347	Level 2
ZEN 3	7.84181363	13.71952964	252	Level 3
DON 1	1.5854636	10.74325581	848	Level 1
DON 2	6.5297169	16.27524666	233	Level 2
DON 3	18.18865713	32.91086986	92	Level 3

**Table 6:** Clustering centers and ranking of three clusters of corn.

Categories	$y_{i}$	${\bar{y}}_{i}$	Sample Size	Quality Level
AFB1 1	2.23192665	4.45638545	604	Level 1
AFB1 2	5.82095175	8.20041504	334	Level 2
AFB1 3	10.01755404	12.27797104	252	Level 3
ZEN 1	15.59706445	28.90062257	482	Level 1
ZEN 2	25.73757556	38.30160156	428	Level 2
ZEN 3	35.83231475	47.86063413	280	Level 3
DON 1	347.19803286	540.86074515	482	Level 1
DON 2	472.40014244	711.26046271	396	Level 2
DON 3	638.92666501	878.91088989	286	Level 3

## Data Availability

The data used to support the findings of this study are available from the corresponding author upon request.
